# Integrating neural organoids and AI: increasing the risk of artificial consciousness or medical malpractice?

**DOI:** 10.3389/fnmol.2026.1767365

**Published:** 2026-03-04

**Authors:** Alexander R. Harris, Patrick McGivern, Kyle C. A. Wedgwood, Frederic Gilbert

**Affiliations:** 1 Department of Biomedical Engineering, The University of Melbourne, Parkville, VIC, Australia; 2 School of Humanities and Social Inquiry, University of Wollongong, Wollongong, NSW, Australia; 3 Living Systems Institute, University of Exeter, Exeter, United Kingdom; 4 School of Humanities and Social Sciences, EthicsLab, University of Tasmania, Hobart, TAS, Australia

**Keywords:** artificial intelligence, clinical trials, ethics, neural organoid, preclinical research, speculative ethics, stem cell

## Abstract

Neural organoids can be integrated with AI models in various formats, termed AI-NO systems. Neural organoids and AI are each high impact research fields which may play significant roles in biological research and clinical decision making. However, their potential benefits, pitfalls and associated governance structures are often overshadowed by highly speculative discourse over the possibility of them developing some form of consciousness and subsequently acquiring a level of moral status. This article examines the cause for this speculative discourse, arguing for a focus on more immediate, empirically grounded ethical issues. It describes potential ethical issues when data obtained from AI models is used for research planning and drug discovery on neural organoids; when AI models are used to analyze data obtained from neural organoids; and when AI models are used to control interventions on neural organoids in open- or closed-loop configurations. It concludes with an investigation on how AI-NO systems may impact clinical decision making.

## 1 Background

Neural organoids can be derived from tissue obtained from healthy people or those with various medical conditions. When derived from human donors, they provide more relevant tissue models for research and clinical applications than can be obtained from animal models. They enable experimentation into the effects for which different genetic and environmental factors have on tissue development and function, and ways of mitigating any adverse behavior. As a result, they are being developed for a range of applications, including for biological research, drug development, personalized drug screening, toxicity testing, phenotypic disease screening and regenerative medicine applications. They can be created in large quantities for pre-clinical research before undertaking any interventions on a person. Neural organoids can also be integrated with AI models (which we term an AI-NO system) which may assist in analyzing the large datasets associated with the applications listed above, and for predicting biological and clinical outcomes. AI-NO systems may enable faster development of more effective clinical interventions, reduce the cost of preclinical research and reduce risk to patients from administering harmful treatments.

Neural organoids are one form of stem cell-derived tissue construct (SCTC), which includes assembloids, induced and bioprinted tissues. The protocols for generating SCTCs are advancing rapidly, allowing creation of larger and more complex tissue structures that better replicate mature tissue (Maisumu et al., 2025). The distinct advantages of neural organoids over other types of SCTCs lie in the unique insights they offer into neural development and neurological disorders. These biological models enable investigations into processes that are otherwise extremely difficult or ethically impermissible to study directly in living fetuses or humans, as doing so would require highly invasive methods with unpredictable and potentially unsafe outcomes.

The development of neural organoids has also led to a related research program sometimes called Organoid Intelligence (OI). OI involves the use of microelectrode arrays to control and record the electrophysiological activity of neural organoids to investigate learning, memory-like phenomena and novel forms of biological computing (Smirnova, 2024; Hartung et al., 2023a,b; Bai et al., 2025; Zafeiriou et al., 2020). The concept of OI closely mimics the development of AI models, raising similar or related ethical and technical issues - including questions about the benefits, limitations, and broader societal implications of advanced AI systems, especially concerns around bias, data ownership, and environmental impact. Neural organoids and AI provide compelling examples for understanding biological systems, models of information processing, and the ethical issues these two technologies can raise, many of which we have investigated in previous work (Walker et al., 2019; Harris et al., 2020, 2022a,b, 2023, 2024, 2025; Walker et al., 2022). Together, these technologies are presented as opening plausible new avenues for investigating cognition and, potentially, certain degrees of consciousness – although the possibility of such developments remains a subject of ongoing debate (Kataoka et al., 2025b; Kataoka and Sawai, 2023; Ishida and Sawai, 2024; Lavazza and Massimini, 2018; de Jongh et al., 2022; Niikawa et al., 2022; Kagan et al., 2022a; Croxford and Bayne, 2024; Van Gyseghem et al., 2025).

The integration of neural organoids and AI raises a variety of new opportunities and ethical issues. AI-NO systems may play a distinct role at each stage of research, development, clinical application and OI. Neural organoid formation involves lengthy, complicated experimental protocols and they generate vast amounts of information, including genetic, epigenetic, phenotypic and treatment response data; and this data must be integrated with patient information, including disease symptoms and biomarkers to achieve useful clinical outcomes. AI models may be able to analyze these very large datasets and detect small or irregular features. An AI-NO system may speed up research and development, and improve clinical decision making by a range of processes: it may help identify relevant biomarkers, identify correlations and distinctions among various SCTCs or population groups, identify new drugs, or reduce clinical errors from application of inappropriate treatments to particular people or groups. Non-clinical applications of AI-NO systems may be to assist in understanding information processing and consciousness or for the development of new computational methods.

This article investigates the potential ways in which AI can be integrated with neural organoids for research and clinical application, and identifies novel technical and ethical issues which may arise from the different types of AI-NO systems. Neural organoids and AI are both hot topics, gaining publicity ranging from recent technical achievements to highly speculative future scenarios, with the more speculative discussions often receiving an overweighted citation rate and level of publicity (Nestor and Wilson, 2025; Kataoka et al., 2023). As such, there is a risk that research on AI-NO systems will also focus on issues such as the emergence of consciousness, higher-order mental properties, or moral status, while ignoring other issues which can impact on SCTCs of all tissue types, not just neural tissue. Therefore, section (1) of this article examines speculative ethical claims in the context of neural organoid research, arguing that concerns about AI-NO consciousness can distort ethical discourse and governance priorities to the detriment of other important issues (Nestor and Wilson, 2025; Hyun et al., 2022; Kataoka et al., 2023). The following sections focus on novel technical and ethical issues that may arise with increasing forms of AI-NO integration which have already been demonstrated or are being openly discussed (Khalifa and Albadawy, 2024; World Health Organisation, 2024; Harris et al., 2023): (2) AI generated data is applied to neural organoids in some way, (3) AI is used to analyze neural organoid derived data, and (4) AI is used to control neural organoid function, including via closed-loop interactions. Broader issues around clinical application of AI-NO systems are then investigated. This article structure enables a systematic investigation of how different forms of AI-NO integration impact system behavior and ethical issues, with the risk of some potential but important repetition across article sections. An alternative article structure examining separate issues across all AI-NO systems risks oversight or domination of certain issues, and would be of less utility to those developing specific AI-NO systems. We emphasize that the article focuses on neural organoids, yet many of the arguments are applicable to other SCTC and tissue types.

## 2 Exaggerated issues associated with integrating neural organoids with AI

Do AI-NO systems constitute a morally urgent domain necessitating distinct analytical focus and precautionary oversight beyond that required for neural organoids in isolation (Kataoka et al., 2025a,b; Sawai et al., 2022)? Ethical and philosophical debates – echoed widely in the media – often center on whether increasing biological and computational complexity in neural organoids might eventually confer morally significant properties such as sentience, phenomenal consciousness, or the capacity to suffer. Some scholars argue that neural organoids raise unique moral concerns not seen in other forms of stem cell research, particularly due to the possibility that they might one day develop consciousness or higher cognitive functions (Koplin and Savulescu, 2019). According to them, these concerns are intensified when organoids are connected to machine learning or AI models that enable real-time interaction, potentially amplifying their cognitive capabilities. Others (Greely, 2021), advocate for a proactive moral framework that balances the immense potential benefits of organoid research with the need to prevent unethical experimentation, emphasizing the importance of defining moral limits now, before organoids reach levels of complexity that could raise serious ethical concerns. Yet, it is important to recognize that the prominence of such ethical debates reflects a broader pattern: across the history of Ethical, Legal and Social Issues (ELSI) research, speculative ethics has often dominated public and academic discourse, (Nordmann, 2007; Hansson, 2020; Caulfield, 2016; Racine et al., 2014; Gilbert and Goddard, 2014) with scenarios ranging from self-replicating nanorobots to optogenetic manipulation of human behavior (Gilbert and Goddard, 2014; Toumey, 2019). These discussions, frequently inspired more by dramatic scenarios than empirical reality, continue to shape how emerging technologies are understood and governed.

These concerns underscore both the promise and pitfalls of neural organoids integrated with AI. However, much of the current discourse surrounding artificial consciousness remains highly speculative. Speculative assertions that AI-NO systems – such as those involving the so called DishBrain – might develop consciousness or morally relevant capacities are, at present, not substantiated by empirical data and risk conflating imaginative speculation with scientific plausibility, (Nestor and Wilson, 2025; Adam, 2025a; Hyun et al., 2022), reinforcing the need for proportionate, evidence-sensitive oversight. This is not to deny that future artificial consciousness scenarios merits academic discussion. Rather, it is to observe that their salience often outpaces the current evidentiary base and risks crowding out governance of ongoing, demonstrable risks (Nestor and Wilson, 2025). Proportionate, evidence-sensitive oversight is therefore warranted. We therefore regard some degrees of speculative ethics as conditionally legitimate and valuable as a complementary–rather than dominant–mode of inquiry: it should map plausible risk envelopes, articulate guardrails, and specify early warning indicators, while remaining explicitly constrained by available evidence and recalibrated to empirical progress (see Table 1). While current evidence does not support consciousness in AI-NO systems, we acknowledge that consciousness related speculative concerns would become empirically actionable were convergent indicators of morally relevant capacities to emerge (Croxford and Bayne, 2024).

**TABLE 1:** Empirical anchoring of risk (Observed → Plausible → Possible).

Biological risks	Evidence now	Anchor example
Cell line mix-ups/contamination	Observed	Longstanding cell-culture errors
Tumor/teratoma risk if transplanted	Observed	Pluripotent stem cell therapy safety literature
Unintended differentiation/plasticity	Observed	Off-target lineage in stem-cell work
Ectopic circuit integration in hosts	Plausible	Neural graft effects in animals (e.g., seizure-like patterns within organoids)
Immunogenicity/rejection (allogeneic)	Observed	Human leukocyte antigen mismatch responses
High batch-to-batch variability	Observed	Organoid variability across labs
**Algorithmic risks**	**Evidence now**	**Anchor example**
Dataset/algorithmic bias	Observed	Medical AI performance gaps
Hallucinated outputs	Observed	LLMs fabricating steps/citations
Black-box opacity	Observed	Low clinician trust/contestability
Distribution shift (out-of-scope data)	Observed	Deployed machine learning drifts across sites
Reward-hacking in closed loops	Plausible	Reinforcement learning systems optimize proxy artifacts
Data rights/privacy/IP disputes	Observed	Secondary use without consent
Cybersecurity/adversarial manipulation	Observed	Medical devices/machine learning attacks
**Clinical risks**	**Evidence now**	**Anchor example**
Automation bias/deskilling	Observed	Clinician over-reliance
Equity of access/benefit sharing	Observed	Health-tech rollouts
**Other risks**	**Evidence now**	**Anchor example**
Artificial consciousness/morally relevant sentience in AI-NO systems	Possible	Public claims around “neurons in a dish” sentience have been criticized and walked back; many neuroscientists argue critical conditions are missing (e.g., embodiment, rich sensory coupling).

Legend: Observed = already seen in adjacent AI/biomedical contexts. Plausible = credible but not yet observed in AI-NO systems. Possible = speculated but no evidence for or against.

In contrast to these speculative ethical concerns, a common neuroscientific viewpoint is that, given our current understanding, consciousness in disembodied neural organoids remains epistemically uncertain and, at present, highly improbable. Many neuroscience articles dismiss concerns regarding neural organoid consciousness due to the absence of critical features such as embodiment, sensory inputs and environmental coupling (Croxford and Bayne, 2024). While we believe these arguments against organoid consciousness are too simplistic and require deeper investigation, we argue here that ethical works should prioritize proportionate, evidence-based oversight, rather than overextend protection to entities with hypothetical properties (Nestor and Wilson, 2025)^1^.

Current discourse also shows a narrow focus on a single neural model –neural organoids which are small, largely unstructured and undeveloped tissue– while overlooking longstanding research with resected neural tissues from animals and humans for research and clinical applications (Wickham et al., 2020); organisms which are generally regarded as having moral status. These resected tissues can be significantly larger than neural organoids, have mature, developed structure and cellular composition, and may have undergone significant memory formation. Yet resected tissues are generally considered to be non-conscious entities and have no moral status. This suggests the concerns around current neural organoids developing consciousness and moral status are, at best, premature –especially given arguments that consciousness in NO is a comparatively low-priority topic relative to more pressing scientific and moral issues (Barnhart and Dierickx, 2023; Van Gyseghem et al., 2025).

In contrast to these more speculative claims regarding the potential for morally significant forms of experience in AI-NO systems, other real and urgent ethical priorities – such as extending legal and regulatory protections to cognitively complex and clearly sentient non-primate animals used in novel and experimental settings – demand immediate attention. Ethical and policy frameworks should be proportionate to the available evidence: rather than pre-emptively legislating for hypothetical AI-NO consciousness, resources should focus on strengthening animal ethics and on developing tools to detect morally relevant capacities in entities we already have good reason to believe can suffer. Pre-emptive legislation of AI-NO systems based on unfounded capacities may undermine trust in their use, result in unjustified moratoriums, prevent development of valuable new technologies which benefit society, and waste time developing unnecessary regulations.

Why, then, does so much ethical attention gravitate toward speculative scenarios, such as AI-NO artificial consciousness? Several interrelated ethical discussions explain the disproportionate attention to speculative scenarios in neural organoid ethics. First, neurohype and media amplification (Wexler, 2019). It is a well-documented pattern in neuroscience communication; institutional press releases and media outlets often amplify early-stage findings through exaggerated claims and superlative language (Gilbert et al., 2018a,b, 2019), while individuals and organizations may seek to gain financial rewards or raise their public profile (Bretzner et al., 2011). This rhetorical phenomenon, neurohype, privileges dramatic possibilities over incremental realities, shaping both research agendas and public appetite for sensationalism. For example, calls to abandon terms such as “mini-brains” or “brain-in-a-dish” when referring to neural organoids reflect concerns about misleading metaphors (Kataoka et al., 2023; Bassil, 2024). Likewise, recent debates over “mind-reading” devices and speculative “neurorights” legislation have tended to fixate on far-fetched scenarios rather than realistic technical capabilities (Gilbert and Russo, 2024a,b). In terms of AI-NO systems, this would mean creating premature or overbroad regulation and protecting the rights of emerging AI-NO entities with capacities that are not currently evidenced and may never materialize (Nestor and Wilson, 2025; Adam, 2025a). Second, the cognitive salience of certain imaginable scenarios. Concepts like “mini-brains that might become conscious” are highly imaginable and narratively compelling, granting them a visibility advantage over diffuse but pressing issues like neural-data governance or procurement protocols (Kataoka et al., 2025a). For instance, the framing of OI as a biocomputing frontier further reinforces this allure, despite its current technical immaturity and uncertainty (in this article, we refrain from the use of OI, rather using more descriptive terms over the form of integration within an AI-NO system). Finally, there exists cross-domain spill over from AI ethics. Organoid ethics debates have increasingly absorbed concerns from broader AI ethics, particularly from discussions around artificial general intelligence and existential risk. This spill over effect risks redirecting ethical scrutiny from current harms (e.g., algorithmic bias, inequitable access, data privacy) to hypothetical future threats, replicating a well-documented problem in AI governance (Mueller, 2024). If these speculative narratives dominate the ethical landscape, we risk consciousness inflation, where scarce regulatory resources and academic attention are monopolized by scenarios with little or no empirical basis. As recent calls in AI ethics have emphasized, governance efforts should refocus on real-world, empirically documented risks. The same imperative applies to neural organoid research: speculative ethics should not dominate discourse.

In contrast to speculative concerns, the integration of AI with neural organoids introduces a set of immediate, empirically grounded ethical challenges that remain underexplored. These include the governance of AI models, vast, high-dimensional datasets generated by organoids and linked patient information (involving a range of data governance issues such as data accuracy, bias and security); the safety and accountability of AI-driven closed-loop systems capable of delivering real-time electrical or pharmacological interventions; and the transparency and auditability of algorithmic decision-making in experimental and clinical contexts. Unlike the hypothetical possibility of the emergence of consciousness, these issues are already material: they affect how AI-NO systems are designed, validated, and deployed today, and they carry direct implications for patient privacy, research integrity, and translational equity. We therefore advocate rebalancing ethical attention: prioritize proportionate, evidence-based oversight of concrete risks while maintaining a watchful, noninflationary posture toward speculative consciousness claims.

## 3 Potential issues when using AI generated data on neural organoids: research and drug discovery

To consider these concrete risks in more detail, we begin by describing two examples where data generated by an AI model informs subsequent work with neural organoids, but there is no direct connection between the two components (subsequent sections discuss examples involving direct data transfer connectivity between both components).

Our first example is where data generated by an AI model is used to assist in neural organoid research planning. This example is reflected across all areas of AI-assisted scientific research (Khalifa and Albadawy, 2024; World Health Organisation, 2024), but has not yet been articulated in the neural organoid field. In this example, the AI model would be trained on protocols and other relevant data detailing neural organoid formation, development, maintenance and analysis from a variety of sources including research articles, guidance documents, patents, and user input. Model training could involve a continual learning process, where new information is provided by the software supplier or via user input. The model could also incorporate relevant ethical, regulatory and scientific guidelines, such as from the International Society for Stem Cell Research (ISSCR) standards (International Society for Stem Cell Research, 2023). This type of AI model could be embedded in electronic lab notebooks to help define and optimize research methods, experimental protocols, analysis and statistics for a researcher. The aim of such a system would be to ensure more neural organoid studies are of high quality, ethically compliant and reproducible. However, the use of AI in this role may raise a range of issues, including the appropriate selection of training methods, protocols and ethical guidelines. For example, the AI models may be trained on and recommend “gold standard” research protocols using the most expensive equipment and materials, or specific commercial products, impacting on less wealthy labs being able to perform these experimental protocols. It may only utilize information from high impact (or open source) journals, ignoring data from more junior or less well known scientists. It may be dominated by large numbers of articles based on research fads or cultures (e.g., favoring particular analytical methods) rather than validated and foundational data. It may also incorporate erroneous or fabricated data and articles which have been retracted. Information entered into the AI model by researchers may also be used to update the model; this could improve the models accuracy or lead to a radicalization toward certain methods. The inclusion of certain ethical standards may tell researchers to follow ethical or regulatory rules/concerns raised from specific groups or jurisdictions which are not applicable to the user, such as avoiding the use of embryonic stem cells, the formation of certain cell types or limit the length of cell culture to minimize the risk of morally significant states from arising.

Even with properly curated training data, the training of AI models may be difficult. Poorly trained models may fail to capture the highly technical language or complex experimental protocols used in stem cell research, and important small differences between protocols; they may fail to identify critical experimental details, instead focussing on commonly used phrases; and they may fail to solve complex mathematical problems (Fang et al., 2025), so there is a non-negligible chance that any statistical information they provide will be incorrect. Model training may never provide sufficient information for every different experimental permutation (e.g., differences in genetic and cellular diversity) and experimental technique (e.g., variations due to systematic errors or sample contamination). Poorly designed, trained or used (e.g., in context learning and users inputting insufficient or inappropriate data) AI models can provide false or misleading information, termed AI hallucinations, resulting in poor research protocols. To limit the risk and impact of AI hallucinations, the models could be required to provide reasoning for a suggested research protocol, so that users can identify incorrect recommendations and adjust or reject the protocols as required. However, even if this provided an adequate method for verifying a model’s reliability, the need to invest additional time and resources in the verification process might detract from the benefits of using AI models in the first place.

An AI model behaving as intended may still result in inappropriate use in research planning. Users may become complacent, failing to recognize errors in protocols and significant details in their experiments. This could result in poor research outcomes, with users having difficulty in justifying their research in publications and preventing others from reproducing their work. Reliance on AI models trained on published research may bias neural organoid research away from novel problems and techniques, leading to further growth in derivative-type publications. This could be further impacted by reviewers utilizing the same AI models, leading to the rejection of articles or grant applications which apply different methods (Adam, 2025b).

The use of AI models in research planning may also lead to complicated intellectual property disputes (Aboy et al., 2025). For example, data provided by a user to train the model may be used by other people or the software provider for their own research planning. Identifying the inventor of a new discovery in these cases may be very difficult or impossible and the legal status of the AI model itself has not been settled. The laws governing AI in research is a complex topic which is only beginning to be investigated (Gaidartzi and Stamatoudi, 2025). New guidelines will need to be developed for consent processes for user data to be used in updating an AI model, and new details on intellectual property ownership will need to be clearly articulated.

Given the abilities and limitations of AI-NO systems in research planning described above, while there is a potential productivity benefit for researchers using AI models to plan or optimize routine neural organoid research, it is likely that there will be less benefit for more innovative research, and a great risk to the quality of research if it is used incorrectly. Ultimately, the appropriate use of AI models in neural organoid research may require such high levels of user oversight that they fail to provide significant advantages.

Our second example is where an AI model is used for drug discovery before screening on neural organoids. In this situation, an AI model may be trained on known drugs and drug targets to predict potential new drug entities. These drugs can then be screened on a neural organoid to assess impact on disease biomarkers and toxicity prior to animal and human trials. However, the quality of the drug predictions are limited by the training of the AI model and the validity of any biomarkers (Harris et al., 2024). To improve the quality of AI-led drug discovery, newer AI models are beginning to include other relevant training data, including Absorption, Distribution, Metabolism, Excretion, and Toxicity (ADMET) information (Fu and Chen, 2025). The difficulty with this approach is in knowing what training data is relevant and ensuring that the data are robust. This is of particular concern for drugs targeting the brain, as the drugs must have the correct physicochemical properties to cross the blood-brain barrier; the brain displays many emergent features which we do not understand how they arise and subsequently don’t know what training data is relevant (Harris et al., 2020); and there are a limited number of drugs and known drug targets which control neural disorders that can be used to train an AI model. Given the enormous complexity of biological systems, it is possible that AI models may never be trained on all of the relevant data, limiting the accuracy of any predictions. Subsequently, neural organoids will play a critical role in validating data obtained from AI models, but there will remain a risk of serious harm or lack of effectiveness when AI-predicted drugs are assessed in a clinical trial.

Of further concern is that AI-led drug discovery would be trained on known interactions and clinical outcomes, so that new drug predictions will most likely identify “me-too” drugs which act in a similar manner to existing drugs. Subsequently, any predicted drugs would not improve outcomes for intractable disorders requiring completely novel interventions, ultimately limiting the utility of AI-led drug discovery. A recent attempt to overcome this issue used an AI model to combine chemical fragments to identify novel antibiotics, but it proposed millions of potential compounds, many of which were unrealistic or could not be easily synthesized (Marchal, 2025). Nevertheless, any promising antibiotics identified by this process still need to be fully investigated to understand their method of action, safety, efficacy, pharmacodynamics and pharmacokinetics. It remains to be seen if drug screening on AI-NO systems (as opposed to neural organoids without AI) will assist researchers significantly in identifying new therapies for treatment refractory patients.

## 4 Potential issues when analyzing neural organoid data with AI models: research and disease screening

Neural organoids generate vast amounts of information which can be analyzed by an AI model, including electrophysiology, ‘omics and structural data, with further integration with genetic and patient-derived and environmental data also possible. AI models can analyze these large datasets and detect small or irregular features. This type of AI-NO system may be used to predict biological and clinical outcomes, including new insights into biological processes and identify potential biomarkers for medical interventions or early disease diagnosis. However, this can come with risks of incorrect detection when using a poorly trained or used AI model.

AI models may be trained on one data type or combine multiple types with different formats, including text, numerical, image and audio formats, and proprietary information. Once again, there are potential issues raised by the training of the AI models which can result in AI hallucinations or misleading information. The training dataset must be from a properly labeled and representative population, as poorly trained models may incorrectly label test data (e.g., healthy biomarkers as unhealthy and vice versa). If the test data contains information not in the training data set, the AI model may generate false information. And while it is hoped that AI models will only detect relevant biomarkers, poor training can result in the detection of irrelevant features, such as user induced effects as part of a testing protocol, or reading user labels rather than measuring the actual sample.

AI hallucinations can also arise from users not providing the AI model with correct or sufficient information in the correct format to make a valid prediction. This may be due to the use of different equipment or data formats used from the AI training data, errors in data curation [e.g., the “vegetative electron microscopy” optical character recognition error (Ford, 2025)], or data obtained from the neural organoids not being large enough or too variable for the AI model to analyze. In fact, it is not clear how an AI-NO system would handle variability and integrate different information. There are multiple sources of variability associated with neural organoids. Neural organoids within a single experiment can display variability due to different genetic clones, cellular composition and structure, resulting in variable behavior and biomarker expression. Information collected by different researchers and facilities/equipment can also vary due to different calibration, bias, systematic errors, analytical methods or reporting methods. Researchers deal with these issues through multiple sample repeats and statistical analyses, including rejection of outliers. It isn’t clear if an AI model would apply the same analytical processes, incorporate all of the data, or reject some data; and it may not be easy to determine what the model has done or why. Regardless of the cause, AI hallucinations causing incorrect or misleading neural organoid analysis can have varying impact, ranging from wasted research efforts, to publishing of false data, and misdiagnosis of patients.

A properly trained AI model can be used to measure biomarkers in test data which have been defined by a user, or to identify new biomarkers (Aerts et al., 2014). However, the measurement of a neural organoid biomarker by an AI model does not imply these features are relevant to the user or connected to humans in any way. Neural organoids are distinct entities in relation to the patients they are derived from, and whose biomarkers must be validated against patient biomarkers and clinical outcome assessments (Harris et al., 2024). The biomarkers measured by an AI model in a neural organoid may be considered validated biomarkers of human disease by professional bodies or regulatory agencies when measured in patients, but they must still be validated in neural organoids, and AI models. Many neural organoid biomarkers measured by an AI model will not be validated biomarkers, rather they will be measurements made by individual researchers, equipment and software suppliers. The validity of these biomarkers may be further limited by the equipment and software available for analyzing neural organoids in comparison to those used to analyze patients (e.g., MRI and CT imaging may be used on patients but may be incompatible with AI-NO systems).

There will be significant data governance issues arising from AI-NO systems used for data analysis relating to data rights. AI developers must gain permission to access all of the training data. Even if people are willing to provide tissue samples for personal diagnostics and treatment screening, they may not be willing to provide information for training an AI model, especially if the model is proprietary.

Finally, the reasoning of why an AI model makes a certain prediction may not be understood by a user. This may confuse researchers when trying to understand what information is useful, exacerbating the big data problem of neural organoids, reducing trust in the results provided by an AI-NO system and ultimately limiting the utility of the information provided. Conversely, leaving all neural organoid analysis to an AI model trained on previous data sets may enhance user complacency, so that novel, interesting data is not identified, reducing the power of AI-NO systems in research. Subsequently, AI-NO systems may provide a significant benefit on the productivity and accuracy of data analysis for research and disease screening platforms, but this will depend on the quality of data provided to it and the validity of the biomarkers it measures.

## 5 Potential issues when controlling neural organoids with an AI model: neural development and OI

Our final example involves an AI model being used to control direct interactions with neural organoids (e.g., controlling a device delivering electrical stimulation or drugs). In this system, the AI model would control the location, timing and type of intervention made to a neural organoid. This type of AI-NO system can be run in an open-loop setup with the AI function controlled by a user or via information in a database; or in a closed-loop setup involving real time measurement and data analysis of neural organoid behavior. The open or closed-loop structure of the AI-NO system and its intended purpose will likely determine the necessary training process of the AI model. When neural organoids are used in research and clinical applications, the precise delivery of chemical and electrical input may be critical to its development and behavior. Therefore, use of an AI-NO system, rather than individuals, to control interventions on neural organoids may better direct tissue development, structure and function, ensuring the desired neural organoid is created and improving reproducibility. In the case of AI-NO systems used for OI type applications, the incorporation of AI into neural organoids may be critical in forming the desired neural structure and information processing.

Many biological factors governing the electrophysiological behavior of neural systems have already been determined, such as neural connectivity, input-output functions, long-term potentiation and long-term depression. However, the rules governing an AI model stimulating a neural system may not be known or even contradict typical neural behavior (e.g., stimulation timings or locations may occur in unexpected patterns). While the AI model supplies some form of intervention, it may not be clear why it is delivering a particular type, timing or location for that intervention. Subsequently, any AI driven intervention may be atypical, resulting in unwanted organoid behavior or development. This could be exacerbated in a closed-loop system, where the AI model is analyzing organoid data which may also be atypical. For example, electrical stimulation induces an artifact which resembles a large action potential (spike). A poorly trained, closed-loop AI-NO system could maximize “spike rate” by repeatedly stimulating the organoid to induce electrical artifacts. If the AI-NO system induces the formation of atypical neural structures, it could alter the organoids behavior in a way which undermines its intended role (e.g., inducing spontaneous seizures); and the organoid may develop novel behaviors which cannot be recognized by a closed-loop AI model.

Another issue is that people using AI-NO systems may fail to understand the systems learning processes, leading to incorrect attribution of system capabilities and properties. An AI-NO system involves two forms of learning, the AI model can update its analysis and control of neural organoid behavior; and the neural organoid can change network structure, synapse connectivity and strength. AI learning (training) is relatively fast, while the different forms of neural organoid learning occur over longer timescales. Differences in learning speed may be a benefit in studying learning processes in AI-NO systems, but could result in a failure of integration between AI and organoid. AI learning may be associated with better detection of specific electrophysiological patterns on a single neural organoid, which can be achieved without any biological learning. However, continual learning of an AI model may result in constant changes to the model in response to an organoids spontaneous activity or background noise. Furthermore, the structure and behavior of organoids are notoriously variable, so that an AI model which has been trained on the behavior of one neural organoid may not be correctly trained to analyze another organoid. Conversely, neural organoid learning can result in long-term changes in behavior, which may be detected by AI models created from a range of training datasets. The risk is that users may misinterpret learning in an AI-NO system as neural organoid learning and memory instead of the less impressive AI learning and memory. Neural organoid learning must be confirmed by applying the same AI model to different neural organoids, and vice versa; and by immunohistochemical and microscopic analysis of neural organoid structure. To our knowledge, only short-term learning processes (a few minutes duration) associated with short-term potentiation/depolarization and long-term potentiation/depolarization have been demonstrated in neural organoids (Zafeiriou et al., 2020), no long-term learning and memory processes associated with changes in neural structure have been shown to date. While previous studies provide important baseline data on the capabilities of an AI-NO system with AI learning, only after long-term neural organoid learning has been demonstrated will the full capabilities of AI-NO systems be ascertained. Researchers claiming current AI-NO systems are learning may not be distinguishing between AI and neural organoid learning and memory, or more worryingly, they may be leaning in to neurohype.

There is great potential and benefit in using AI to control neural organoids. More detailed analysis of the capabilities and ethical issues of this application will depend on the research outcomes published over the coming years.

## 6 Potential issues arising from clinical application of neural organoids integrated with AI models: personalized drug and disease screening

It is expected that neural organoids will play an increasing role in clinical decision making, particularly for personalized drug screening and phenotypic disease screening. In these applications, cells obtained from individual patients are converted into neural organoids with phenotypic features compared to control organoids for disease diagnosis, while changes in phenotypic features after exposure to different drugs would be used for high throughput drug screening. We have previously investigated some of the ethical and regulatory issues related to these clinical applications (Walker et al., 2019, 2022; Harris et al., 2020, 2022b,^2023^, ^2025^). As discussed above, the integration of AI into analysis of neural organoid data may have significant benefits in dealing with large datasets, and incorporation of other relevant data. For example, the AI model could integrate geographical, population and lifestyle information to provide more accurate and patient specific diagnostics or treatment recommendations, or more rapidly identify rarer diseases that may otherwise be missed or slow to diagnose by healthcare workers. However, poorly trained and used AI models may provide inaccurate or misleading information. This section examines some of the unique issues an AI-NO system could raise when used for clinical decision making^2^.

In the clinical application of AI-NO systems, inappropriate AI training and hallucinations could result in significant health implications through misdiagnosis and incorrect drug recommendations. This would also reduce trust in the technology. Subsequently, the training requirements of the AI model would be stricter than for non-clinical applications of AI-NO systems. Training should be based on validated biomarkers obtained from the most recent clinical knowledge and accepted diagnostic criteria from professional bodies. This would prevent AI-NO systems recommending proprietary treatments, under/over diagnosing conditions or providing information biased by religious or political views. Furthermore, the AI model should only be updated after appropriate validation processes have occurred; it should not undergo user-based continual learning, which may introduce biases or errors. The downside to using a fixed AI model is that it may prevent optimization of clinical outcomes for certain groups, although this may be at the expense of other people (Sparrow et al., 2024). A fixed model would also lag behind clinical research so that diagnoses and treatments based on the latest clinical knowledge would require other diagnostic methods. An alternative strategy could be to allow continual learning of the AI model and classifying its use as medical research and any patients as research subjects (Sparrow et al., 2024). However, research subjects, as opposed to patients, are not expected to receive benefit from a clinical intervention; and an AI model trained on one individual may not generate data which is considered generalizable knowledge which is applicable to other patients.

While the use of AI-NO systems may help detect rarer diseases, there may be less data on these diseases for training the AI model. Various co-morbidities and emerging conditions (e.g., novel infections, diseases, toxic contamination) may also not be included in training data and subsequently incapable of being identified by the AI model. As a result, AI-NO systems may fail to deliver significant benefits in diagnosing rare, complex and emerging diseases. The lack of training data or novel behavior outside of its training data, may also enhance the risk of AI hallucinations. In the case that AI hallucinations do occur, it is critical that AI-NO systems convey an “I do not know” response, rather than providing an incorrect diagnosis or drug recommendation.

The regulation of AI-NO systems for clinical use will be complicated and impacted by jurisdiction (Angus et al., 2025). The decision to classify AI-NO systems as either a medical device or research tool will have dramatic implications on how the technology is regulated, who maintains oversight, patient informed consent requirements and health insurance coverage (Sparrow et al., 2024). Diagnostic devices, including companion diagnostics, have varying levels of regulatory oversight which can depend on its accuracy and intended use. Meanwhile, regulations over AI are yet to be created for many jurisdictions. The European Union has only recently created the EU AI Act, a world-first legal framework governing all AI use within the EU. The Act classifies AI models according to risk (unacceptable, high or minimal), with medical devices utilizing AI being considered high-risk. However, even in the EU, the oversight of AI-NO systems under current regulations may not be sufficient, as the classification of AI-NO systems as medical devices is unclear, traditional evaluation methods of medical devices are poorly matched to AI, and safeguards have not yet been implemented (Ong et al., 2025). Regulatory approval of the organoid component will require validation of its biomarker expression against patient biomarkers, symptoms and clinical outcome assessments (Harris et al., 2024). The AI component will likely require a different form of validation, which ensures the model is running as intended and accurately measures the organoid biomarkers. However, the validation process may be prevented by a range of reasons, including inconsistencies between patients, poor measurement of patient biomarkers, symptoms and clinical outcome assessments. Critically, any changes to the organoid or AI model would require full system revalidation. To ensure safe clinical application of AI-NO systems and so that trust is built for its use, there must be an international organization to push uniform standards and governance of AI in clinical practice (Ong et al., 2025).

Once approved for use, the processes for integrating AI-NO systems into clinical decision making will need to be developed. AI-NO systems used for personalized drug screening may provide a large list of potentially suitable drugs with no other information to assist clinicians in choosing the most appropriate drug for a patient, requiring complex decision processes. A clinician could incorporate other patient information into the decision-making process after receiving the results from the AI-NO system, alternatively they could provide information to the AI-NO system to shorten the drug list or identify an optimum drug candidate. This information could be provided to the AI model before or after the drug screening process has occurred. The information could include a range of patient data, such as lifestyle choices, co-morbidities and impact of potential side effects. However, the choice of information provided by a clinician may be biased, leading to suboptimal clinical outcomes or entrenchment of biases already present in the healthcare system. The system may also have limited or no capacity to weigh up moral judgments or understand the precautionary principle, only recommending drugs with a maximum impact on an organoids phenotypic behavior. There will need to be very clear, standardized user input into the AI model to ensure it provides appropriate clinical information. It may be necessary to run clinical trials to determine the most appropriate method for integrating AI-NO systems into clinical decision making.

The use of AI-NO systems in clinical decision making may also impact on clinician behavior. Clinicians may be reluctant to diagnose patients by phenotypic screening on SCTCs, as the biomarkers may have no visible relationship with patient biomarkers and symptoms. Patient diagnosis with an AI-NO system may be even more difficult, as the AI model may be detecting very obscure biomarkers. Furthermore, as current AI models do not provide explanation or justification, clinicians may not understand its drug recommendations or diagnoses, and be hesitant to use this information (Hatherley et al., 2024). This has even led to arguments that it is more important for AI predictions to be interpretable rather than accurate, as clinicians are able to inspect information flagged by the AI model and integrate other types of information for clinical decision making (Hatherley et al., 2024). Conversely, clinicians may feel financial, social or time pressures to transfer decision making over to AI-NO systems (Goddard et al., 2012). In the event that clinicians become over-reliant on the technology, it could lead to cognitive deskilling, where clinicians reduce their expertise or lose confidence in their abilities. This could result in an over-valuing of data provided by the AI model, even against their own judgments, and increase the risk of patient harms (Goddard et al., 2012). This will require an educational process for clinicians and pathology lab workers to understand the capabilities and limitations of AI-NO systems.

There should be debate around the appropriate response for when a patient receives an incorrect diagnosis or treatment based on data obtained from an AI-NO system. This should determine who has responsibility over each step of AI-NO use, examine existing malpractice frameworks to determine if they are suitable, and provide guidance on how pathology labs and clinicians detect errors and adjust a patients’ healthcare. It should also create guidelines and procedures to determine who is liable in the case of clinical errors (e.g., clinicians, pathology lab technicians, biochemical, hardware and software suppliers). Other relevant organizations should also be consulted to determine if they understand the capabilities and limitations of AI-NO systems, and if the data the systems provide is sufficient for the organization to perform its role in the provision of patient healthcare (e.g., regulators, human research ethics committees, insurance companies).

Finally, the development of AI-NO systems requires significant expertise, lab facilities and data storage, this could result in a stratification of AI-NO system access to more affluent jurisdictions and conditions more common in those jurisdictions. Efforts will be needed to ensure AI-NO systems are made available to poorer communities and diseases they are affected by. The complex validation processes, may also result in jurisdictions with less developed health care systems using AI-NO systems for clinical applications without the necessary validation, AI training and health care worker education. An education campaign will be needed to ensure AI-NO systems are only used after appropriate validation has been undertaken.

## 7 Conclusion

AI-NO systems may assist in biological research, drug development, personalized drug screening, toxicity testing, phenotypic disease screening and regenerative medicine applications, and play a role in understanding learning, memory formation and biological computing. These benefits may be achieved by recommending optimal and ethical research protocols, identifying new drug entities, analyzing large datasets, identifying relevant biomarkers, and controlling interventions to direct organoid structure and function. However, the combination of two high impact research fields, neural organoids and AI, risks fomenting highly speculative discourse around the formation of conscious entities with moral status, demanding immediate regulatory protections. This may lead to pre-emptive legislation of AI-NO systems based on unfounded capacities, undermining trust in the technology, an unjustified moratorium on their development and use, prevention of the development of valuable new technologies which benefit society, and wasted time developing unnecessary regulations. We argue that ethical discussions around AI-NO systems should be based on more immediate, empirically grounded issues and recommend clear governance actions (for some examples see Table 2).

**TABLE 2:** Where AI meets organoids → what can go wrong → what to do.

AI-NO integration	Main risk (one-liner)	Main governance action
AI helps plan experiments (e.g., smart assistants)	Wrong or biased steps creep into protocols	Require sources + human sign-off for each suggested step
AI suggests new drugs → then screen on organoids	“Me-too” drugs or false positives from weak biomarkers	Pre-specify validation; only move forward if human biomarkers align
AI analyses organoid data (e.g., ‘omics/imaging)	Mislabels or chases artifacts; privacy conflicts	Use representative datasets; log decisions; allow “I don’t know” outputs
AI controls stimulation/drug delivery (open-loop)	Unsafe timing/dose patterns	Bound parameters; safety interlocks; full activity logs
AI controls in real time (closed-loop)	“Rewards-hacks” artifacts (e.g., boosts spike artifacts)	Multi-metric objectives + artifact filters; safe algorithm development
Clinical decision support using AI-NO systems	Misdiagnosis; automation bias; unclear liability	Locked/validated models; clinician in the loop; incident reporting

The development of useful AI-NO systems is dependent on their training protocol and use. To prevent AI hallucinations, all relevant data must be included in training an AI model and users must input appropriate data.

AI-NO systems used in research planning may increase user productivity involving routine neural organoid research, but be of less benefit for more innovative research protocols. It may also lead to complicated intellectual property disputes which are only in the early stages of being addressed by legal scholars. The use of AI models in neural organoid research may require such high levels of user oversight that they fail to provide significant advantages.

Artificial intelligence-led drug discovery has potential to identify many new therapies, but given the enormous complexity of biological systems, it is possible that AI models may never be trained on all the relevant data, limiting the accuracy of any predictions. Subsequently, neural organoids will be critical in validating data obtained from AI models. However, the training of AI models on known drugs and drug targets will likely identify me-too drugs which act in a similar manner to existing drugs. Subsequently, AI-predicted drugs may not improve outcomes for intractable disorders requiring completely novel interventions, ultimately limiting the utility of AI-led drug discovery.

AI models may be able to analyze large data sets obtained from neural organoids. However, neural organoids are notoriously variable, and it isn’t yet clear how an AI model would handle this variable information to provide useful data. Organoids are also distinct entities in relation to the people they are derived from. Subsequently, organoid biomarkers must be validated against patient biomarkers and clinical outcome assessments (Harris et al., 2024). Many neural organoid biomarkers measured by an AI model will not be validated biomarkers. This may impact on users understanding and appropriate use of the data provided by AI-NO systems.

AI models may be important tools in controlling interventions on neural organoids to ensure appropriate development and function. This can be implemented in open- or closed-loop configurations. These systems will involve two forms of learning associated with the AI model and the neural organoid. There is a risk that uses will fail to understand the systems learning processes, leading to incorrect attribution of system capabilities and properties.

Finally, AI-NO systems may play various roles in clinical decision making. Poorly trained AI models may entrench bias in medicine or lead to patient harms. AI-NO systems may also modify clinician behaviors in ways that undermine the potential benefits of the technology. It is critical that AI-NO systems used in clinical decision making are validated properly and that the users, be they pathology labs or clinicians, understand the systems capabilities and limitations. However, the classification and relevant regulations of AI-NO systems have not yet been developed. The decisions on how to classify AI-NO systems will have dramatic implications on how the technology is regulated, who maintains oversight, patient informed consent requirements and health insurance coverage.

## Data Availability

The original contributions presented in this study are included in this article/supplementary material, further inquiries can be directed to the corresponding author.

## Footnotes

1 Even less conceptually demanding notions such as *sentience* illustrate this point. The description of neural organoid–AI behavior as “sentient” by Cortical Labs (Kagan et al., 2022b) was strongly criticized by the field as scientifically unwarranted (Balci et al., 2023). Tellingly, the lead author of Kagan et al. (2022b) later acknowledged this in *Nature*, stating: “I wouldn’t use the word sentient going forward” (Adam, 2025a).

2 We note that in these examples, a pathology lab would be running the AI-NO system, with information provided to and from a clinician. It is unlikely a clinician would be in direct contact with the AI-NO system.
